# Maximizing SNR per unit time in diffusion MRI with multiband T‐Hex spirals

**DOI:** 10.1002/mrm.29953

**Published:** 2023-12-29

**Authors:** Maria Engel, Lars Mueller, André Döring, Maryam Afzali, Derek K. Jones

**Affiliations:** ^1^ Cardiff University Brain Research Imaging Centre (CUBRIC) Cardiff University Cardiff UK; ^2^ Leeds Institute of Cardiovascular and Metabolic Medicine University of Leeds Leeds UK

**Keywords:** diffusion MRI, magnetic field monitoring, multiband, simultaneous multi‐slice, spiral imaging

## Abstract

**Purpose:**

The characterization of tissue microstructure using diffusion MRI (dMRI) signals is rapidly evolving, with increasing sophistication of signal representations and microstructure models. However, this progress often requires signals to be acquired with very high *b*‐values (e.g., *b* > 30 ms/μm^2^), along many directions, and using multiple *b*‐values, leading to long scan times and extremely low SNR in dMRI images. The purpose of this work is to boost the SNR efficiency of dMRI by combining three particularly efficient spatial encoding techniques and utilizing a high‐performance gradient system (*G*_max_ ≤ 300 mT/m) for efficient diffusion encoding.

**Methods:**

Spiral readouts, multiband imaging, and sampling on tilted hexagonal grids (T‐Hex) are combined and implemented on a 3T MRI system with ultra‐strong gradients. Image reconstruction is performed through an iterative cg‐SENSE algorithm incorporating static off‐resonance distributions and field dynamics as measured with an NMR field camera. Additionally, T‐Hex multiband is combined with a more conventional EPI‐readout and compared with state‐of‐the‐art blipped‐CAIPIRINHA sampling. The advantage of the proposed approach is furthermore investigated for clinically available gradient performance and diffusion kurtosis imaging.

**Results:**

High fidelity in vivo images with *b*‐values up to 40 ms/μm^2^ are obtained. The approach provides superior SNR efficiency over other state‐of‐the‐art multiband diffusion readout schemes.

**Conclusion:**

The demonstrated gains hold promise for the widespread dissemination of advanced microstructural scans, especially in clinical populations.

## 1 INTRODUCTION

The current trend towards ever more intricate models of tissue microstructure calls for diffusion MRI (dMRI) with a multitude of different contrasts, including multiple diffusion directions,^1^, ^2^ multiple diffusion weightings,^3^, ^4^ and/or several TEs.^5^, ^6^, ^7^ Moreover, many approaches demand the use of very high diffusion weightings (e.g.,^8^, ^9^, ^10^), which in turn lead to very low SNR and the need for multiple averages. All of this results in cumbersome and often lengthy (>1 h) experiments.^11^, ^12^, ^13^, ^14^

To render the experiments feasible for patient populations that are unable to remain still for a long period of time (and, therefore, to mitigate adverse effects from subject motion) it is vital to reduce scan time. The two main solutions from an acquisition perspective are: (i) minimizing the number of required contrasts for the given research question^15^, ^16^, ^17^, ^18^; and (ii) minimizing the number of repetitions of each contrast encoding instance, that is, organizing the spatial encoding as efficiently as possible. The latter is the focus of this work.

Spiral k‐space trajectories offer efficient spatial encoding by making very good use of the gradient system.^19^, ^20^, ^21^, ^22^ Furthermore, in a spin‐echo experiment, spirals achieve the shortest possible TE by acquiring the k‐space centre at the start of the readout, which boosts the otherwise low SNR in dMRI experiments compared to a standard rectilinear EPI readout.^23^ To date, spiral dMRI has been performed using consecutive slice‐by‐slice imaging and k‐space sampling^24^, ^25^, ^26^, ^27^ in 2D. To leverage the additional encoding capabilities of the receive coils in the third dimension, 3D^28^ or multiband (MB)^29^ imaging can be used.

Recently, we proposed that a particularly time‐efficient 3D acquisition could be obtained by tilting of the hexagonal grid (T‐Hex) underlying 3D k‐space for stacked trajectories.^30^ However, with the exception of very low spatial resolutions, 3D imaging requires multiple shots to acquire the entire k‐space. This is unfavorable for dMRI, where shot‐to‐shot motion can lead to severe image artifacts^31^ and steady‐state conditions favor longer repetition times. Instead, in this work, we propose to combine T‐Hex sampling with MB imaging, allowing for time‐efficient single‐shot dMRI. Moreover, we use spiral trajectories for efficient in‐plane encoding and to utilize the additional SNR it offers. On the hardware side, we deploy a strong gradient system^26^, ^32^, ^33^ (up to 297 mT/m amplitude) for efficient diffusion encoding. Lastly, to obtain maximum image fidelity, we use an expanded signal model for the image reconstruction.^34^ This includes field dynamics,^35^ monitored with a dedicated camera, as well as static field inhomogeneities and coil sensitivities.

## 2 METHODS

### 2.1 Trajectory design

In general, spatial encoding of N simultaneously excited slices spaced ∆z apart from each other requires sampling of a k‐space slab of thickness $\frac{2\pi }{∆z}$.^36^ As in conventional 2D and 3D encoding, the sampling density is determined by the FOV, where now ${\mathrm{FOV}}_{z}=N∆z$, and by the desired undersampling factor R in case of parallel imaging.^37^ Thus, compared to full 3D encoding, the MB encoding differs solely in that it requires a lower resolution in the *z* direction, namely ∆z, rather than the actual image resolution required in *z* direction, which is instead determined by the bandwidth of the excitation RF pulse and the associated slice selection gradient amplitude. For T‐Hex sampling,^30^ originally devised for 3D encoding, the amendment for MB imaging is straightforward as the resolution in the *z* direction can easily be controlled by the number of shots and the slab‐thickness in k‐space (d in^30^) of each individual shot. Just like for the 3D case, T‐Hex sampling for MB can be combined with different in‐plane sampling strategies, most importantly spirals and EPI.

### 2.2 High *b* ‐value diffusion weighted imaging

Scans of two healthy volunteers (female, both age 34) were performed on a 3T Connectom scanner (Siemens Healthcare GmbH, Erlangen, Germany) using a 32‐channel receive array (same vendor). Ethical approval was attained from the Institutional Review Board of the Cardiff University School of Psychology and informed consent was obtained from the participants.

**FIGURE 1:**
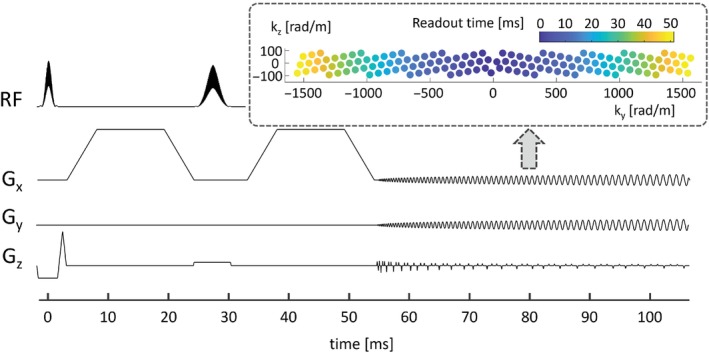
Sequence diagram. For the maximum *b*‐value of 40 ms/μm^2^, *G*_*x*_ = 297 mT/m is reached. Note that the *G*_*z*_ axis is zoomed by a factor of 5 to discern the small blips during the T‐Hex readout. The inlay shows the T‐Hex sampling scheme in k‐space (central cross‐section).

The dMRI sequence (Figure 1) comprised phase‐optimized excitation and refocusing pulses,^38^, ^39^, ^40^ a pulsed‐gradient spin echo encoding,^41^ and T‐Hex spiral readouts (generating vector $\vec{v}=\left[2,1\right]$).^30^ FOV = 20.4 × 20.4 × 10.2 cm^3^, 2 mm isotropic resolution, 3 simultaneously excited slices, 51 slices in total, overall undersampling factor *R* = 2.2, 51.8 ms readout duration, TE = 54.6 ms, TR = 2.08 s. Diffusion weighting with *b* = 0.5, 1, 2, 3, 5, 7, 10, 14, 19, 25, 32, 40 ms/μm^2^ was applied along six diffusion directions (+*x*, −*x*, +*y*, −*y*, +*z*, and −*z* in scanner coordinates) in randomized order (cf. Figure 3) with diffusion gradient spacing, Δ, and gradient duration, δ, of 29.93 and 16.14 ms, respectively. The maximum gradient amplitude and slew rate was 297 T/m and 60 T/m/s, respectively. The latter was constrained by the vendor's peripheral nerve stimulation (PNS) model. For the readout, the slew‐rate limit was relaxed to 185 T/m/s. The complete acquisition (which included six initial dummy scans and regularly‐interspersed *b* = 0 ms/μm^2^ scans) took 3 min 7 s.

### 2.3 Image reconstruction

Field dynamics were measured with a dedicated camera (Skope Magnetic Resonance Technologies, Zurich, Switzerland) and incorporated up to third order, including second order concomitant fields,^25^, ^42^, ^43^ in the image reconstruction, which was based on a cg‐SENSE^44^ algorithm including off‐resonance correction^30^, ^34^ (commercial version “skope‐i”). The platform provided by the scanner vendor does not allow the usually applied model‐based eddy current corrections (ECC)^45^, ^46^, ^47^ to be controlled separately for transmit and receive mode. Therefore, the ECC for both transmit and receive was applied and the B_0_ term of these corrections was demodulated from the coil data (receive) before image reconstruction.^48^ This is necessary as the field camera is ignorant of such corrections and any B_0_ fluctuations will already be included in the model (zero‐th order spherical harmonics). Coil sensitivity and B_0_ maps were computed from a Cartesian multi‐echo gradient echo (GRE) pre‐scan.

ADC maps were computed from the diffusion weightings with |*b*| < 1 ms/μm^2^.

To assess the quality improvement from incorporating dynamic field information in the image reconstruction for the system and sequence in question, reconstructions of the data from the experiments described in the previous section were also performed including only lower orders of spherical harmonics (up to first and second order), without the second order concomitant fields, and based on the nominal k‐space trajectory (i.e., not using the field dynamics measured with the field camera at all). In this latter case, the model based B_0_ ECC of the system was retained.

The geometric fidelity of the images acquired with strong diffusion weighting was assessed by overlaying white‐matter boundaries obtained from a segmented *T*_1_‐weighted scan^49^ using the Unified NeuroImaging Quality Control Toolbox (UniQC^50^, ^51^), and SPM12 (Wellcome Centre for Human Neuroimaging, London, UK, http://www.fil.ion.ucl.ac.uk/spm/).

### 2.4 SNR comparison

Whereas the SNR gains of spiral imaging^23^ and multiband imaging^52^, ^53^, ^54^ have been quantified previously we aim to study here the SNR gain achieved through the T‐Hex sampling scheme. To this end, different sampling schemes for spin‐echo EPI were implemented and measured in vivo (Table 1a).

**TABLE 1:** Imaging parameters for (a) the SNR comparison, (b) sampling pattern comparison study, and (c) diffusion kurtosis imaging. For (b), TE is omitted as the comparison only comprises the sampling patterns without embedding in an actual sequence. The acquisition time is computed for the same gradient amplitude and slew rate constraints as used in the SNR comparison; however, it is grayed out since the resulting sampling grids depend only on the other parameters. The same holds true for the in‐plane resolution. Diffusion gradient spacing Δ and duration δ are given additionally for (c).

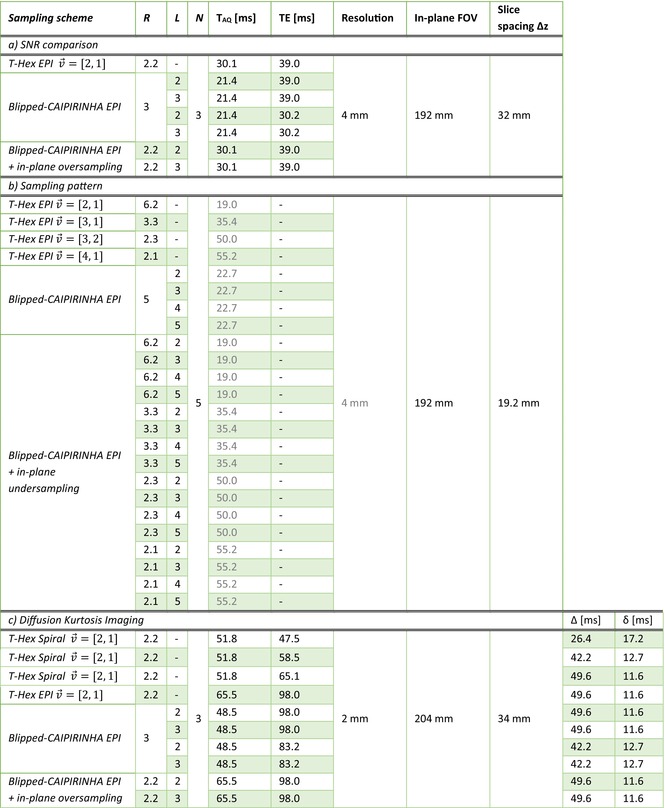

To date, the most widespread technique to accomplish single‐shot MB imaging of N simultaneously excited slices is the blipped‐CAIPIRINHA^32^ approach, where the k‐space sampling is distributed on L distinct k‐space planes in the through‐plane direction with L≤N and a spacing of $∆k=\frac{2\pi }{L∆z}$ between these k‐space planes. In this approach, R≥N.

The SNR comparison was performed for three simultaneously excited slices with 4 mm in‐plane resolution, 19.2 cm FOV and ∆z=3.2cm inter‐slice spacing. T‐Hex with generating vector $\vec{v}=\left[2,1\right]$ (R=2.2) was compared to blipped‐CAIPIRINHA with L=2 and L=3 and R=N, keeping all other sequence parameters fixed. Since the EPI readout train was longer for the T‐Hex case than for the blipped‐CAIPIRINHA case (R=2.2≤N=3), two further comparisons were performed. First, the blipped‐CAIPIRINHA acquisitions were repeated with the minimum TE (30.2 ms instead of 39.0 ms). Second, the blipped‐CAIPIRINHA acquisitions were combined with in‐plane oversampling to achieve the same readout length as the T‐Hex scheme in question, that is, to isolate any effect due to averaging.

Note that EPI was chosen as the in‐plane encoding strategy as it is the most wide‐spread sampling scheme, in particular in the context of multiband imaging.

SNR maps and the respective gain of the T‐Hex scheme were calculated as described in Ref. 23. Synthetic complex‐valued Gaussian noise of the same covariance as measured in separate noise scans, was added to the raw coil data before image reconstruction. Subsequently the standard deviation over 200 such noise corrupted images (complex and magnitude) was computed, and SNR maps were computed as the ratio between an image without added noise and these SDs.^55^

Since the motivation to target sampling on hexagonal grids (or “controlled aliasing”) is to achieve an optimum conditioning of the image reconstruction by closest packing of aliases in the image domain, the (circle) packing density^56^ was computed for all investigated oblique lattices exhibited by the described trajectories. The derivation of this computation for blipped‐CAIPIRINHA sampling as well as corresponding Matlab (MathWorks, Natick, MA) code, including derivation of the optimum value of L is provided as Supporting Information (Figure S1 and accompanying text).

To gain a more complete perspective of how sampling patterns compare, the latter was repeated for an imaging scenario with five simultaneously excited slices with ∆z=1.92cm and, otherwise, the same imaging parameters as described above (Table 1b).

### 2.5 Diffusion kurtosis imaging

To assess the use of multiband T‐Hex spirals on more widely available hardware, experiments with lower *b*‐values and gradient specifications typically found on a clinical system were performed (max amplitude/slew‐rate = 80 mT/m, 100 T/m/s, respectively). The diffusion encoding scheme comprised weightings along 15 non‐collinear directions^16^ on three shells with *b*‐values of 0.8, 1.8 and 2.8 ms/μm^2^, as well as 9 *b* = 0 ms/μm^2^ volumes. The spatial encoding was performed for FOV = 20.4 × 20.4 × 10.2 cm^3^, 2 mm isotropic resolution, 3 simultaneously excited slices, 51 slices in total. With TR = 2.5 s, the acquisition time, including six initial dummy scans, was 2.5 min. The experiment was performed with different readouts and diffusion timings as detailed in Table 1c. A series expansion including both the Gaussian diffusion tensor and diffusion kurtosis tensor was fitted to the magnitude images^57^ as well as a scalar diffusion kurtosis representation, using the powder‐averaged data.^4^

## 3 RESULTS

### 3.1 High *b* ‐value diffusion‐weighted imaging

**FIGURE 2:**
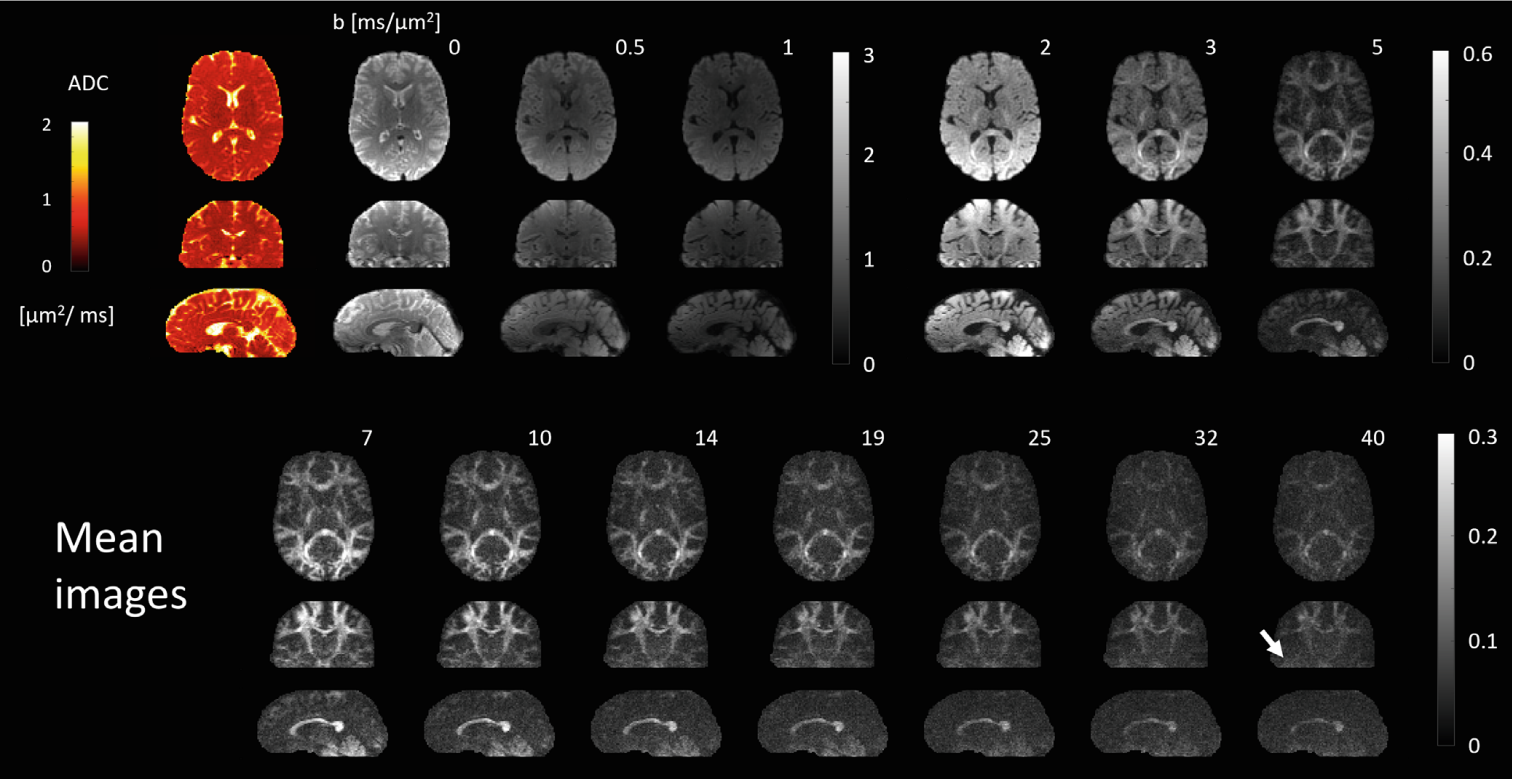
T‐Hex MB spiral dMRI in‐vivo, mean over six diffusion encoding volumes in +*x*, +*y*, +*z*, −*x*, −*y*, and −*z* direction for exemplary transversal, coronal, and sagittal cross‐sections. For visualization purposes, low (0–1 ms/μm^2^), medium (2–5 ms/μm^2^) and high (7–40 ms/μm^2^) diffusion weightings are scaled differently (color scale to the right of each group). In the upper row, the left panel shows the ADC map as computed from the three lowest diffusion weightings (*b* = 0, 0.5, 1 ms/μm^2^). The white arrow points in the highest diffusion weighting (*b* = 40 ms/μm^2^) to a region where noise amplification occurs due to bad conditioning of the image reconstruction.

Figure 2 shows the results from the dMRI experiment with high *b*‐values, namely the mean over the six directions per diffusion‐weighting. The images exhibit an overall high visual fidelity. Noise amplification occurs only in places of steep off‐resonance changes which lead to distorted k‐space encoding^58^ and degrade the conditioning of the image reconstruction (as can be seen in the images with highest diffusion weighting for example, near the ear canals in the coronal view, Figure 2, white arrow). The image reconstruction of these data using all higher‐order field dynamics took ˜18 s per diffusion volume.

Figure S2 details exemplarily the individual diffusion‐weighted images. Notably the SNR suffices to see remaining signal (e.g., in the corpus callosum) and clearly discernible WM/GM boundaries up to the highest *b*‐value without averaging. This is confirmed on the one hand by the geometric congruency between the remaining signal and a white‐matter mask (Gif S1) and on the other hand by the relative signal decay curve of a single exemplary voxel in the cortical white matter (upper right corner of Figure S2), showing remaining signal at the highest *b*‐value of 40 ms/μm^2^ (along the *x*‐axis). Other brain regions (signal decay of an exemplary voxel in the corpus callosum depicted in the lower right corner), are, however, more affected by physiological noise and/or g‐factor noise amplification.

**FIGURE 3:**
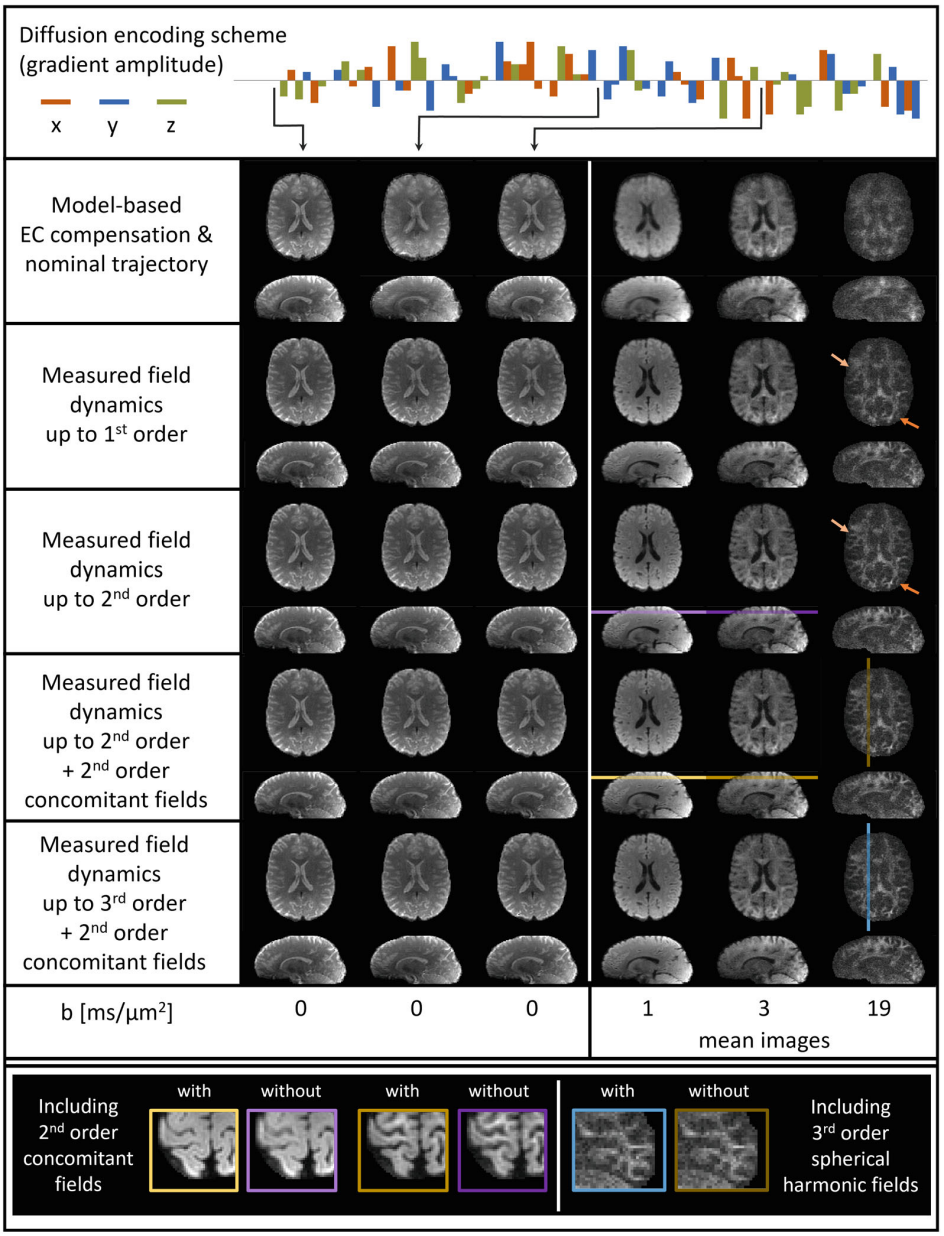
The uppermost row shows the diffusion scheme of the experiment, exhibiting randomly shuffled diffusion weighting in three orthogonal directions and of inverse polarity with varying strength. The second row shows multiband T‐Hex spiral images reconstructed using the nominal k‐space trajectory and the vendor's correction of B_0_ eddy‐currents (demodulation of the raw coil data according to a multi‐exponential model). The third to sixth row show results from the same scan when considering in the image reconstruction field dynamics up to first, second order spherical harmonics, second order concomitant fields, and third order spherical harmonics respectively as measured in a separate experiment with a field camera. For each case, the left panel displays three exemplary b_0_ volumes that were interspersed between the diffusion weighted scans as indicated by the black arrows. The right panel contains the mean images over exemplary diffusion‐weighted volumes with (from left to right) *b* = 1, 3, and 19 ms/μm^2^, realized with ∓47, ∓81, and ∓205 mT/m diffusion gradient amplitudes, respectively. Note that the gray scale was adapted per *b*‐value for improved depiction. The light and dark orange arrows point exemplarily to places where white matter features are lost when only considering field dynamics up to first order. The bottom row depicts on the left side excerpts of peripheral slices (indicated by colors) from the *b* = 1 ms/μm^2^ (light coloring) and 3 ms/μm^2^ (dark coloring) volumes for the reconstructions including field dynamics up to second order spherical harmonics (purple) and additionally including second order concomitant fields (yellow). In the same manner, on the right side, excerpts of a sagittal section from the b = 19 ms/μm^2^ volumes demonstrate the impact of including additionally the third order sphercial harmonic fields (blue).

Figure 3 investigates the significance of accurate measurement of field dynamics and their incorporation into the image reconstruction. For initial *b* = 0 ms/μm^2^ volumes (e.g., here on the leftmost side) the image quality from reconstructions with the nominal trajectory and the vendor's model‐based ECC is only hampered by slight shading artifacts and duplicated edges. However, diffusion‐weighted imaging volumes as well as interspersed non‐diffusion‐weighted volumes exhibit strong, locally varying blurring artifacts. None of these artifacts are retained when performing an image reconstruction based on the measured field dynamics. The bulk of artifacts is already cleaned up when incorporating field dynamics up to first order. However, residual blurring can be observed, especially in the diffusion‐weighted volumes. Inclusion of the second order field dynamics provides noticeable improvement, for example, sharper depiction of the cortical white‐matter tracts in the *b* = 19 ms/μm^2^ volume. A close‐up inspection of peripheral regions (lowest panel in Figure 3) reveals the residual blurring that is removed when incorporating additionally second order concomitant fields (left) and third order spherical harmonics (right). The individual gyri are demarcated more clearly, and the gray‐white matter contrast becomes more distinct.

### 3.2 SNR comparison

**FIGURE 4:**
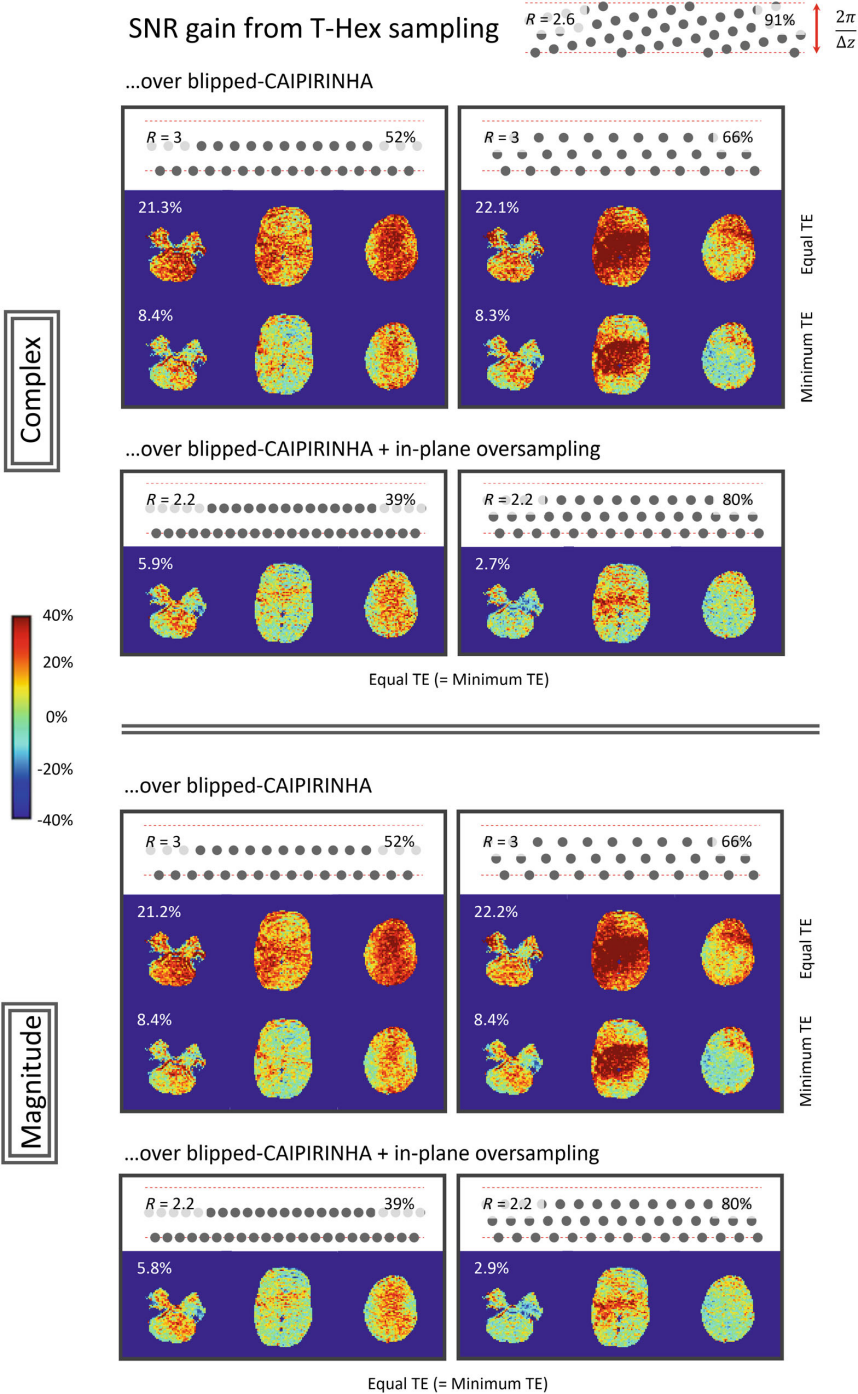
Alternative sampling patterns were compared in their SNR performance to a T‐Hex pattern (upper right corner). Different k‐space trajectories are displayed as excerpts from the sampling pattern in the phase‐encoding plane, that is, each gray dot stands for one frequency encoding line. Dashed red lines delineate the upper and lower bound of the k‐space slab that needs to be sampled to spatially encode the three simultaneously excited slices, each gray dot stands for one frequency‐encoding line. For each of the sampling patterns studied, the packing density of the oblique lattice is displayed in the upper right corner whereas the overall undersampling factor is displayed in the upper left corner. Maps show the percentage SNR gain of T‐Hex when compared to the pattern in question and the mean gain over all three slices is displayed in white numbers in the upper left corner. The upper and lower sections show gains in complex and magnitude SNR respectively.

Figure 4 displays the results of the SNR comparison. As expected, the SNR yield of the individual sampling strategy corresponds with the overall undersampling factor and with TE. Additionally, it correlates with the packing density of the sampling grid. The average SNR over the three simultaneously encoded slices for a T‐Hex sampling is higher than for all other sampling strategies. The SNR gain can become small (down to 3% in this case) when the blipped‐CAIPRINHA scheme is combined with over sampling and the choice of L is taken such that lattices close to a hexagonal grid are reached. These findings are comparable for both magnitude and complex SNR.

**FIGURE 5:**
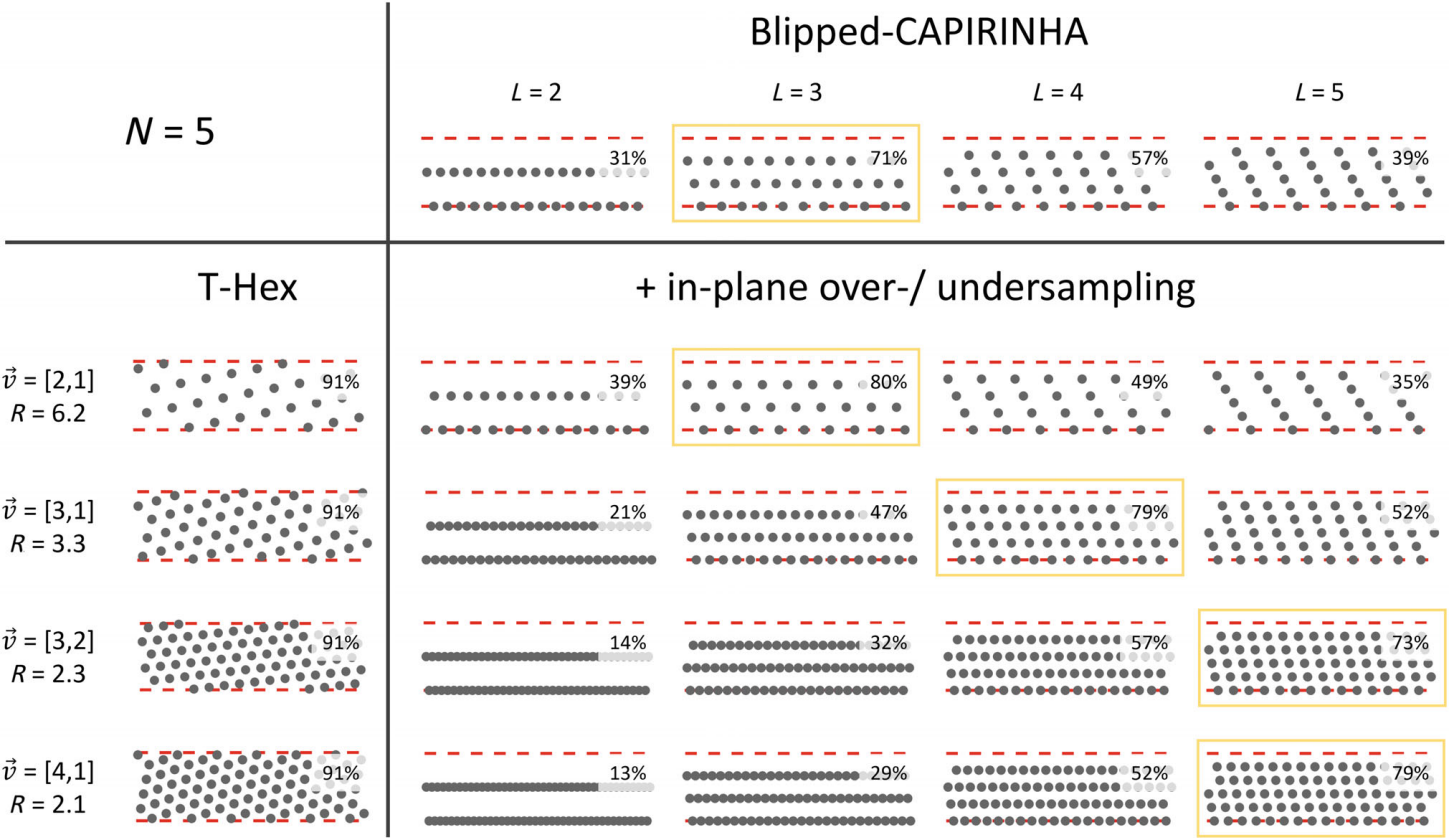
Different k‐space trajectories are displayed as excerpts from the sampling pattern in the phase‐encoding plane, that is, each gray dot stands for one frequency encoding line. Dashed red lines delineate the upper and lower bound of the k‐space slab of thickness 2π/Δz that needs to be sampled to spatially encode the five simultaneously excited slices. The upper row shows four different blipped‐CAIPIRINHA patterns for five simultaneously excited slices with imaging parameters as described in the text and Table 1b. *L* denotes the number of distinct k‐space steps in through‐plane direction. For simplicity, *L* = 1 was left out, as it is hardly used in practice. The four lower rows show on the left side four different T‐Hex sampling patterns resulting in different undersampling factors. For each of these, on the right side, blipped‐CAIPIRINHA patterns with an adapted in‐plane over‐ or undersampling to match the total readout time of the respective T‐Hex pattern, are displayed. In the upper right corner of each pattern the packing density of the respective oblique grid is displayed. For each undersampling factor, a yellow framing indicates the non‐T‐Hex pattern with the highest packing density.

Figure 5 shows the comparison of sampling patterns for five simultaneously excited slices. The T‐Hex patterns offer the highest packing density, which none of the other patterns reach. Among the blipped‐CAIPIRINHA patterns, the value of L that maximizes the packing density (highlighted by the yellow frames) varies, depending on the in‐plane phase‐encoding spacing.

### 3.3 Diffusion kurtosis imaging

**FIGURE 6:**
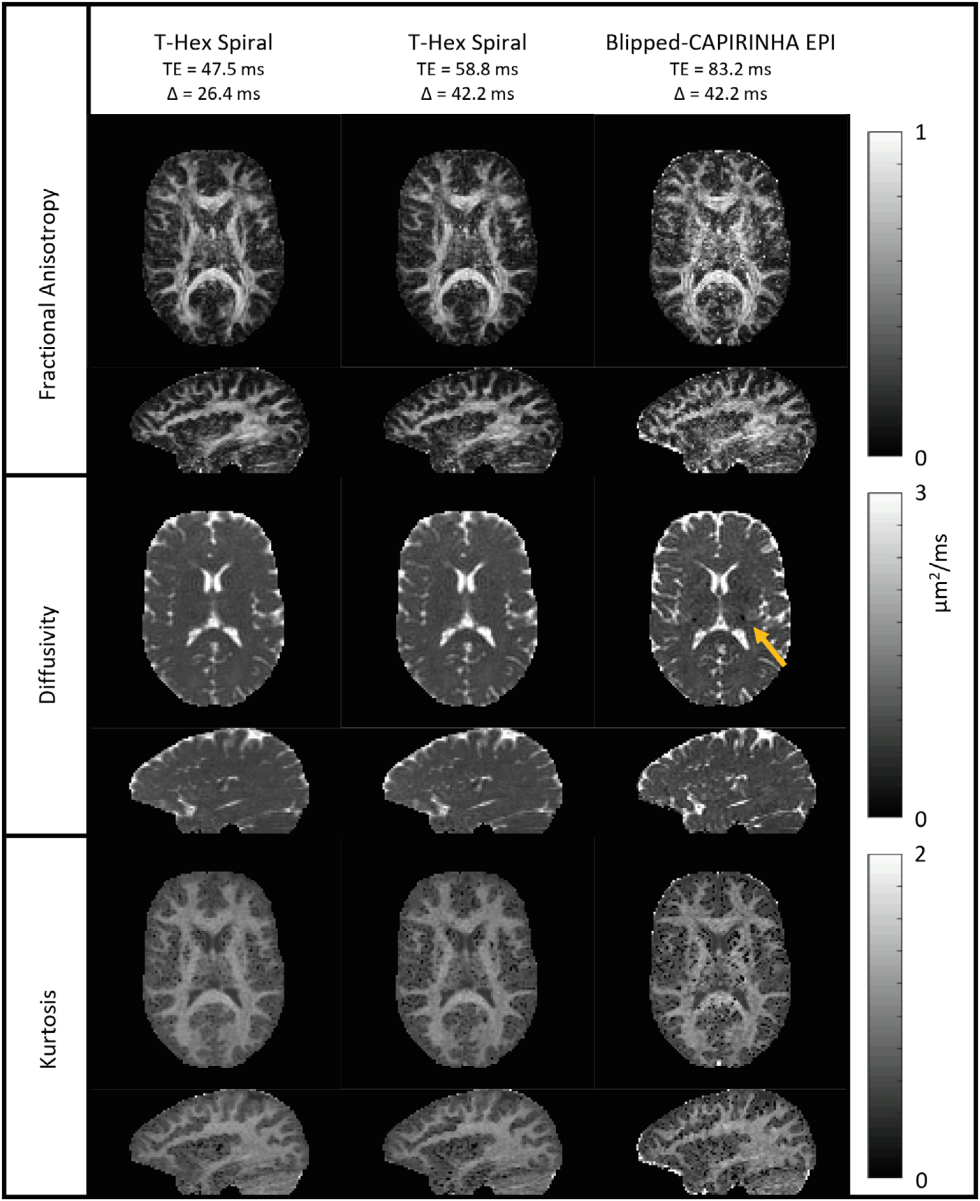
The uppermost row shows maps of fractional anisotropy as obtained from a diffusion kurtosis tensor fit. The second and third row show maps of diffusivity and kurtosis respectively as obtained from the respective fit of the powder‐averaged data. The leftmost and rightmost column contain the results of T‐Hex spiral readout trains and blipped‐CAIPIRINHA EPI, respectively, in each case exhibiting the shortest feasible TE. As this goes along with varying diffusion time Δ, the central column shows the T‐Hex spiral readout with Δ matching the one used for blipped‐CAIPIRINHA EPI and accordingly longer TE. The yellow arrow points to an irregularity in the diffusivity.

Figure 6 shows the key results from the experiments described in Section 3.5, the comparison between the currently most commonly used blipped‐CAIPIRNHA EPI (using L=3, which is optimal according to our findings in Supporting Information S1) and the proposed T‐Hex spiral sampling. All three parameter maps exhibit more noise when using the blipped‐CAIPIRINHA EPI readout than when using the T‐Hex spiral readouts. In the powder‐averaged kurtosis, the noise increase manifests in an increase of “black voxels” (very low kurtosis values indicating that the fit did not converge). The T‐Hex spiral with the shorter TE and shorter diffusion time exhibits the least amount of noise and highest image quality. Figures S3‐S8 contain the complete comparisons. In general, irregularities such as the non‐flat powder diffusivity in white matter indicated in Figure 6 can be observed in the parameter maps derived from EPI‐based experiments in different locations throughout the brain.

## 4 DISCUSSION

We successfully demonstrated MB spiral imaging, enhanced by a T‐Hex sampling scheme. We showed its application in dMRI where it improves the SNR efficiency of the acquisition – SNR maximization being crucial for precision and accuracy of the parameter estimates^59^, ^60^, ^61^ in microstructural modeling.

As shown in Figure 3, accurate knowledge of the field evolution including higher‐order terms is key to faithful image reconstruction in the given dMRI scenario, in agreement with previous findings on different MR systems.^25^, ^48^ The performance of the vendor's ECC combined with nominal trajectories was particularly poor for diffusion encoded volumes but interspersed non‐diffusion‐weighted (*b* = 0 ms/μm^2^) volumes were also affected. This points to long‐term eddy currents as one potential confounding factor.

In the setup used for the current work, due to the nature of the field camera in use, measurements of the field dynamics had to be performed after the actual scanning, thereby effectively doubling the required scan time. This, however, can be tackled readily with cameras designed for concurrent field monitoring^62^ or one‐time calibration‐based gradient impulse response modeling.^63^, ^64^, ^65^

The key aim of T‐Hex is to minimize the number of shots needed, by maximizing k‐space coverage per shot. This is particularly relevant for dMRI with high *b*‐values and long diffusion or mixing times,^66^ which incur a large time overhead per shot.^30^ However, the original 3D T‐Hex still required multiple shots per volume, which can lead to phase‐related artifacts in dMRI. Previous works corrected these artifacts using navigators^67^, ^68^ or the data themselves,^69^, ^70^ requiring additional time overhead per shot or only allowing for limited undersampling. In turn, this limits the achievable acceleration which is one of the key objectives and use cases of T‐Hex. Therefore, T‐Hex dMRI becomes only practicable with the MB single‐shot version presented in this work.

When comparing MB T‐Hex sampling to the current state‐of‐the‐art MB sampling strategy, that is, blipped‐CAIPIRINHA,^32^ two aspects must be considered separately. First, the ability to freely choose the in‐plane phase‐encoding sampling allows, in particular, for in‐plane *over*sampling and thereby decoupling of the number of simultaneously excited slices (N) from the total undersampling factor (R). This has not been done for blipped‐CAIPIRINHA so far but is a natural by‐product of T‐Hex. Second, the ability to tailor the sampling to a hexagonal grid allows for optimal conditioning of the image reconstruction problem assuming an elliptical object and spherically distributed receive coils. This is always fulfilled for T‐Hex, but almost never for blipped‐CAIPIRINHA. Note here, that non‐isotropic sampling requirements (e.g., when the FOV is the same along all axes, but the coil array exhibits distinct sensitivities along only one direction) can be accommodated with T‐Hex by stretching or compressing the tilted hexagonal grid accordingly.

The 22% SNR increase from T‐Hex demonstrated here when taking both aspects together, could lead to a factor of 1.5 fewer averages required and a 15‐min scan could be cut down to 10 min. Adding the 40% SNR gain achieved with spirals,^23^ a 29‐min scan could be cut down to 10 min.

We note that while in‐plane oversampling has not previously been reported with blipped‐CAIPIRINHA, we show in Figure 4 that if it is performed, then for specific imaging scenarios, that is, combinations of FOV, R, N, and ∆z, and for some specific choices of L, sampling patterns close to a hexagonal grid can be achieved with blipped‐CAIPIRINHA (see yellow framings in Figure 5). Furthermore, such oblique lattices correspond to a characteristic tiling of aliases in image space, which might, combined with the given object and coil sensitivity profile, by chance be preferable over hexagonal tiling. However, to our knowledge, there has been no systematic study identifying the value of L for optimal conditioning of the image reconstruction. Moreover, the SNR penalty for non‐ideal sampling is expected to increase for higher undersampling factors.^71^

Our findings corroborate that the T‐Hex ansatz of assuming optimum image reconstruction conditioning to be reached with hexagonal sampling is reasonable for typical brain scan scenarios. Notably, this optimal conditioning is achieved while maintaining smooth *T*_2_* weighting throughout k‐space. The choice of the T‐Hex sampling pattern (generating vector) is subject to a careful balance between readout duration (decisive for the degree of *T*_2_* filtering) and SNR, as is the case for 2D sampling schemes such as EPI or spiral, when the undersampling factor *R* is chosen.

While the SNR comparisons performed here were done based on EPI as the in‐plane encoding strategy, they are expected to directly translate to spirals, which can be combined in the same way with either T‐Hex sampling or blipped CAIPIRINHA sampling,^36^ but offer additional SNR gains due to shorter echo‐times and more efficient usage of the gradient system as well as a more homogeneous sampling.^23^ Blipped‐CAIPIRINHA can, in some cases, achieve a shorter TE than T‐Hex when using an EPI readout, conferring an SNR advantage. However, this SNR advantage does not apply to spiral readouts, as the TE is independent of the readout‐length, with the gradient/spin echo formed at the start of the readout train.

The strict optimization of the k‐space sampling in a controlled way, including also the initially mentioned independent choice of *N* and *R* is paramount, especially for experiments with strong diffusion weighting that operate on the verge of the noise floor and are time‐critical due to the need for a multitude of different diffusion weightings. Here, recovering signal by optimum k‐space sampling rather than averaging is vital to minimize the g‐factor penalty on the one hand and to minimize the repetition of lengthy diffusion encoding modules on the other. Although we did this work on a scanner with high gradient amplitude (300 mT/m), it is equally applicable to machines with lower gradient strength, as currently found in clinical settings. In this context, the T‐Hex sampling scheme might even offer greater benefits at a given *b*‐value, as the overhead per shot is larger with lower gradient amplitudes, if the diffusion module takes longer and the minimization of the number of shots required to cover a given imaging volume becomes more beneficial. The results from the diffusion kurtosis imaging experiments demonstrate the superior image quality reached with T‐Hex spirals over conventional readout schemes using blipped‐CAIPIRINHA. The irregularities observed in parameter maps derived from EPI‐based experiments occur most likely due to spatially dependent g‐factor noise amplification, which shapes up more prominently in Cartesian sampling than in spirals and is bound to appear in different areas depending on the sampling scheme in the phase‐encoding plane.^23^, ^37^, ^44^

Finally, we note that the MB single‐shot version of T‐Hex can be applied to other contrasts such as BOLD fMRI.^72^ There, it might facilitate slice‐timing correction,^73^, ^74^ which is not feasible in multi‐shot 3D imaging with the traditional approach, operating in image space, because each reconstructed voxel contains information from the entire k‐space, that is, from the entire acquisition time required to encode one 3D volume. However, compared to the 3D version of T‐Hex, MB T‐Hex comes at the expense of longer RF pulses and increased SAR levels. Furthermore, it should be noted that regarding the spatial encoding, the feasible accelerations for 3D T‐Hex and MB T‐Hex are approximately equal and are dictated by the object in question and the coil configuration. Hence, the MB approach is preferable to regular 3D imaging only when shot‐to‐shot inconsistencies favor single‐shot acquisition or steady‐state considerations favor longer TR (as in the dMRI application presented here).

Future work should investigate the integration with more time‐efficient^40^, ^75^ and yet off‐resonance‐robust MB pulses and subsequently examine the performance of the new method in expansive dMRI experiments. The gain in SNR when translated to acquisition time advantage holds promise in particular for the application of advanced microstructural models in patient populations that are unable to lie still for a long time.

## Supporting information


**Figure S1.** Packing density computation for blipped CAIPIRINHA sampling.
**Figure S2.** Left panel: GRE scan. Centre panels: T‐Hex MB spiral dMRI along *x* direction (without averaging). Right panel: signal decay for the six diffusion directions +*x*, +*y*, +*z*, −*x*, −*y* and −*z* for two exemplary voxels in the cortical white matter (upper row) and in the corpus callosum (lower row), as indicated with red squares in the central panel. Note that for improved perceptibility, the squares are in each direction a factor 4 larger than the actual voxel.
**Figure S3.** Maps of fractional anisotropy for an exemplary transversal slice as obtained from the DKI experiments.
**Figure S4.** Maps of powder average diffusivity for an exemplary transversal slice as obtained from the DKI experiments.
**Figure S5.** Maps of powder average kurtosis for an exemplary transversal slice as obtained from the DKI experiments.
**Figure S6.** Maps of fractional anisotropy for an exemplary sagittal slice as obtained from the DKI experiments.
**Figure S7.** Maps of powder average diffusivity for an exemplary sagittal slice as obtained from the DKI experiments.
**Figure S8.** Maps of powder average kurtosis for an exemplary sagittal slice as obtained from the DKI experiments.

**Gif S1.** This animation shows the same images as depicted in the rightmost panel in Figure S2 (gray‐scale). The overlay (yellow) is a white matter mask as extracted from an anatomic reference scan.

## Data Availability

The full raw data set of the high *b*‐value experiments (section 2.2/3.1) is openly available in ISMRMRD format^76^ under https://owncloud.cubric.cf.ac.uk (Username: *MultibandSpiralDiffusionMRI* Password: *CUBRIC23!*).
