# Supplementary material for: Maximizing SNR per unit time in diffusion MRI with multiband T‐Hex spirals
Source: Magn Reson Med. 2023 Dec 29;91(4):1323–36. doi: 10.1002/mrm.29953 (PMC10953427; doi:10.1002/mrm.29953)
Supplement: Supplementary file 1 — Figure S1. Packing density computation for blipped CAIPIRINHA sampling. Figure S2. Left panel: GRE scan. Centre panels: T‐Hex MB spiral dMRI along x direction (without averaging). Right panel: signal decay for the six diffusion directions +x, +y, +z, −x, −y and −z for two exemplary voxels in the cortical white matter (upper row) and in the corpus callosum (lower row), as indicated with red squares in the central panel. Note that for improved perceptibility, the squares are in each direction a factor 4 larger than the actual voxel. Figure S3. Maps of fractional anisotropy for an exemplary transversal slice as obtained from the DKI experiments. Figure S4. Maps of powder average diffusivity for an exemplary transversal slice as obtained from the DKI experiments. Figure S5. Maps of powder average kurtosis for an exemplary transversal slice as obtained from the DKI experiments. Figure S6. Maps of fractional anisotropy for an exemplary sagittal slice as obtained from the DKI experiments. Figure S7. Maps of powder average diffusivity for an exemplary sagittal slice as obtained from the DKI experiments. Figure S8. Maps of powder average kurtosis for an exemplary sagittal slice as obtained from the DKI experiments. [file MRM-91-1323-s002.docx]

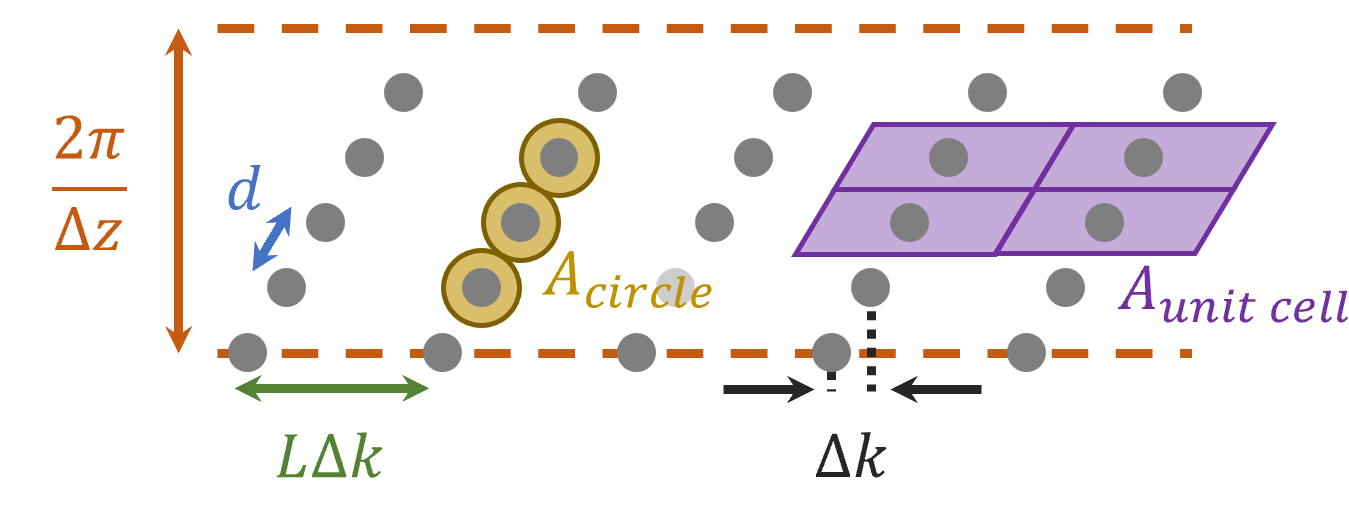


**Figure S1:** Packing density computation for blipped CAIPIRINHA sampling. The packing density $D$ of an oblique lattice is the fraction of the surface covered, if a circle is centered around each lattice point and all circles are of the same dimension and as large as possible without generating overlap. It can be computed as the ratio of the area of one of such circles (yellow) to the area of a primitive unit cell (purple) of the lattice:

|  | $D=\frac{A_{circle}}{A_{unit cell}}$ | (1) |
| --- | --- | --- |

For blipped CAIPRINHA, the area of the primitive unit cell is independent of the choice of $L$ and given as

|  | $A_{unit cell}=\frac{2\pi\Delta k}{\Delta z}$ | (2) |
| --- | --- | --- |

with the in-plane phase-encoding spacing

|  | $\Delta k=\frac{2\pi R_{in-plane}}{{FOV}_{in-plane}}$ | (3) |
| --- | --- | --- |

and $R_{in-plane}$ denoting the in-plane undersampling factor, ${FOV}_{in-plane}$ the in-plane field of view, and $\Delta z$ the distance between simultaneously excited slices.

The area of the circle depends on the orientation of the lattice:

|  | $A_{circle}=\pi r^{2} with r=\min\left( d,L\Delta k \right)/2$ | (4) |
| --- | --- | --- |

where $d$ is the smallest distance between chronologically adjacent k-space lines (blue) and given as

|  | $d=\sqrt{\left( \frac{2\pi}{L\Delta z} \right)^{2}+{\Delta k}^{2}}$ | (5) |
| --- | --- | --- |

Note that the sketch shows a case where $L=5$ and $d<L\Delta k$. Inserting Equations (2) to (5) into Equation (1) yields

|  | $D=\left\{ \begin{aligned} \frac{\pi}{4}\left( \frac{{FOV}_{in-plane}}{L^{2}\Delta zR_{in-plane}}+\frac{\Delta zR_{in-plane}}{{FOV}_{in-plane}} \right) if d<L\Delta k \\ \frac{\pi L^{2}\Delta zR_{in-plane}}{4{FOV}_{in-plane}} otherwise \end{aligned} \right.$ | (6) |
| --- | --- | --- |

Attached Matlab code (ComputePackingDensity.m) computes the optimum choice of $L$ for given imaging parameters according to the packing density derived from Equation (6).


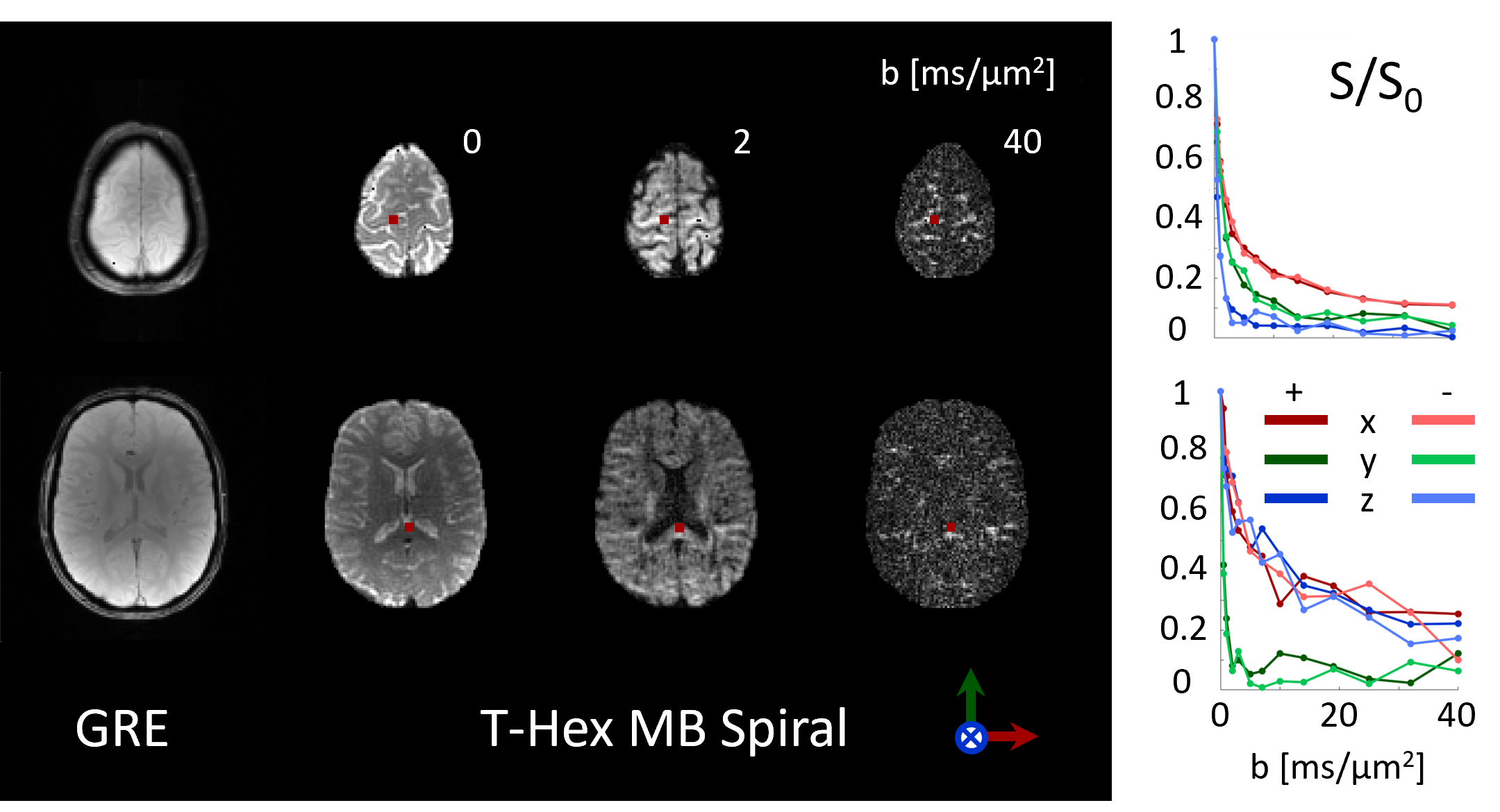


**Figure S2**: Left panel: GRE scan. Centre panels: T-Hex MB spiral dMRI along x direction (without averaging). Right panel: signal decay for the six diffusion directions +x, +y, +z, -x, -y and -z for two exemplary voxels in the cortical white matter (upper row) and in the corpus callosum (lower row), as indicated with red squares in the central panel. Note that for improved perceptibility, the squares are in each direction a factor 4 larger than the actual voxel.


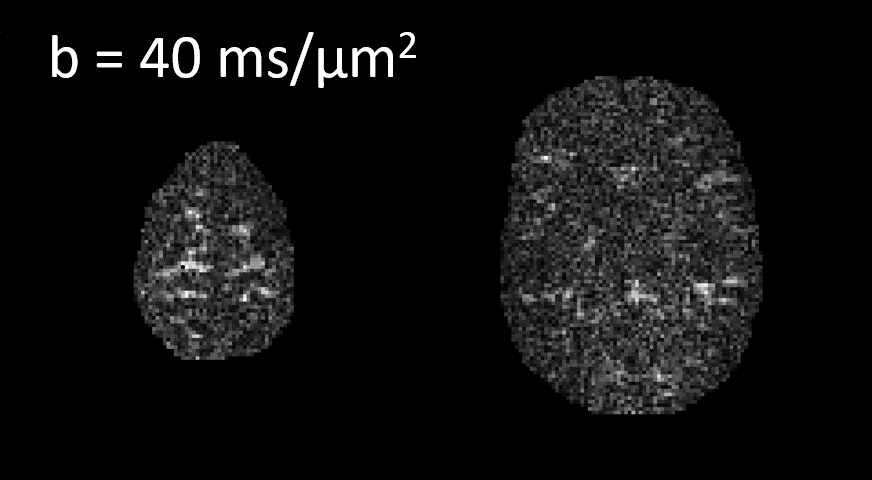


**Gif S1:** This animation shows the same images as depicted in the rightmost panel in Figure S2 (grey-scale). The overlay (yellow) is a white matter mask as extracted from an anatomic reference scan.


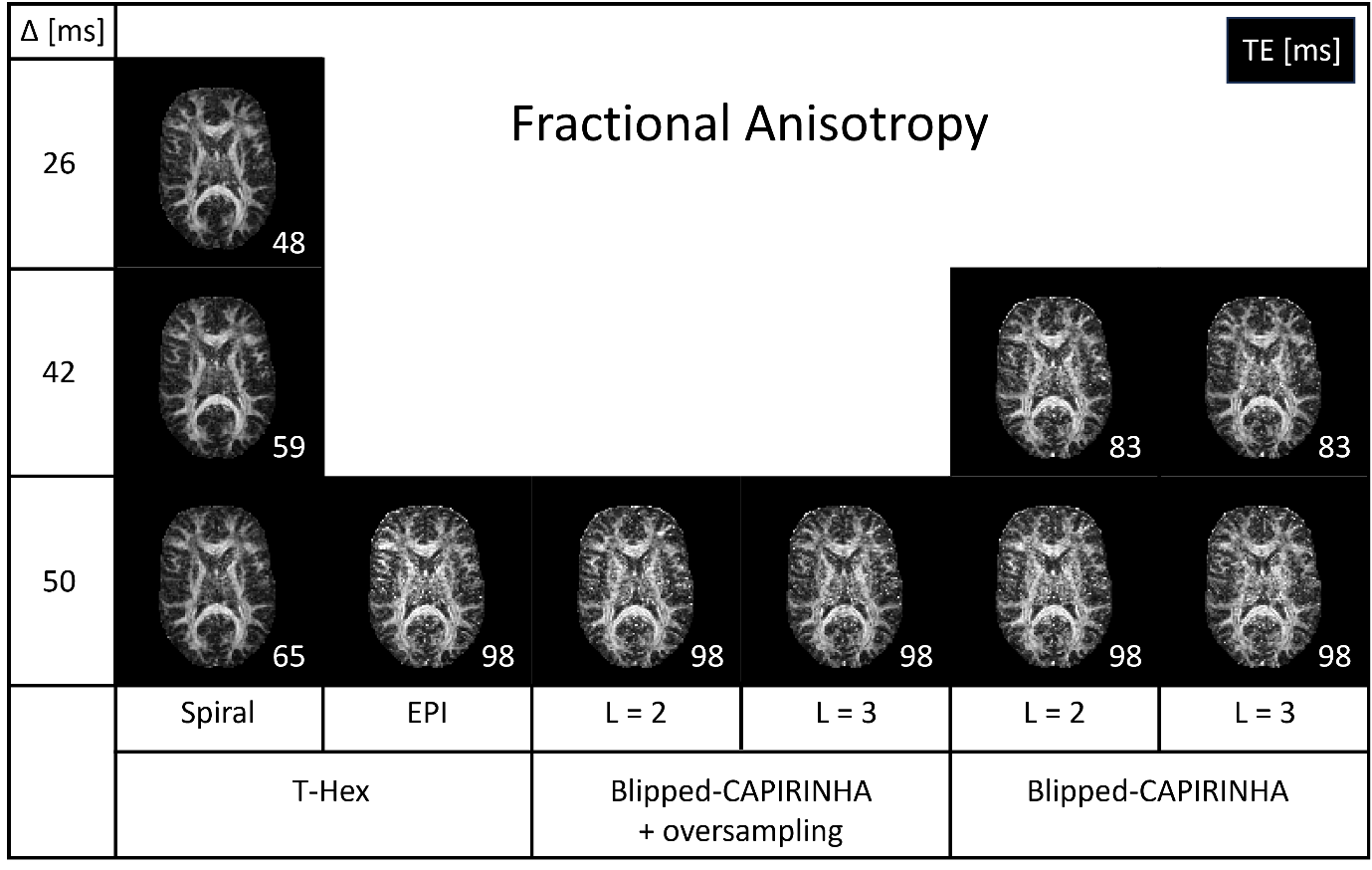


**Figure S3:** Maps of fractional anisotropy for an exemplary transversal slice as obtained from the DKI experiments.


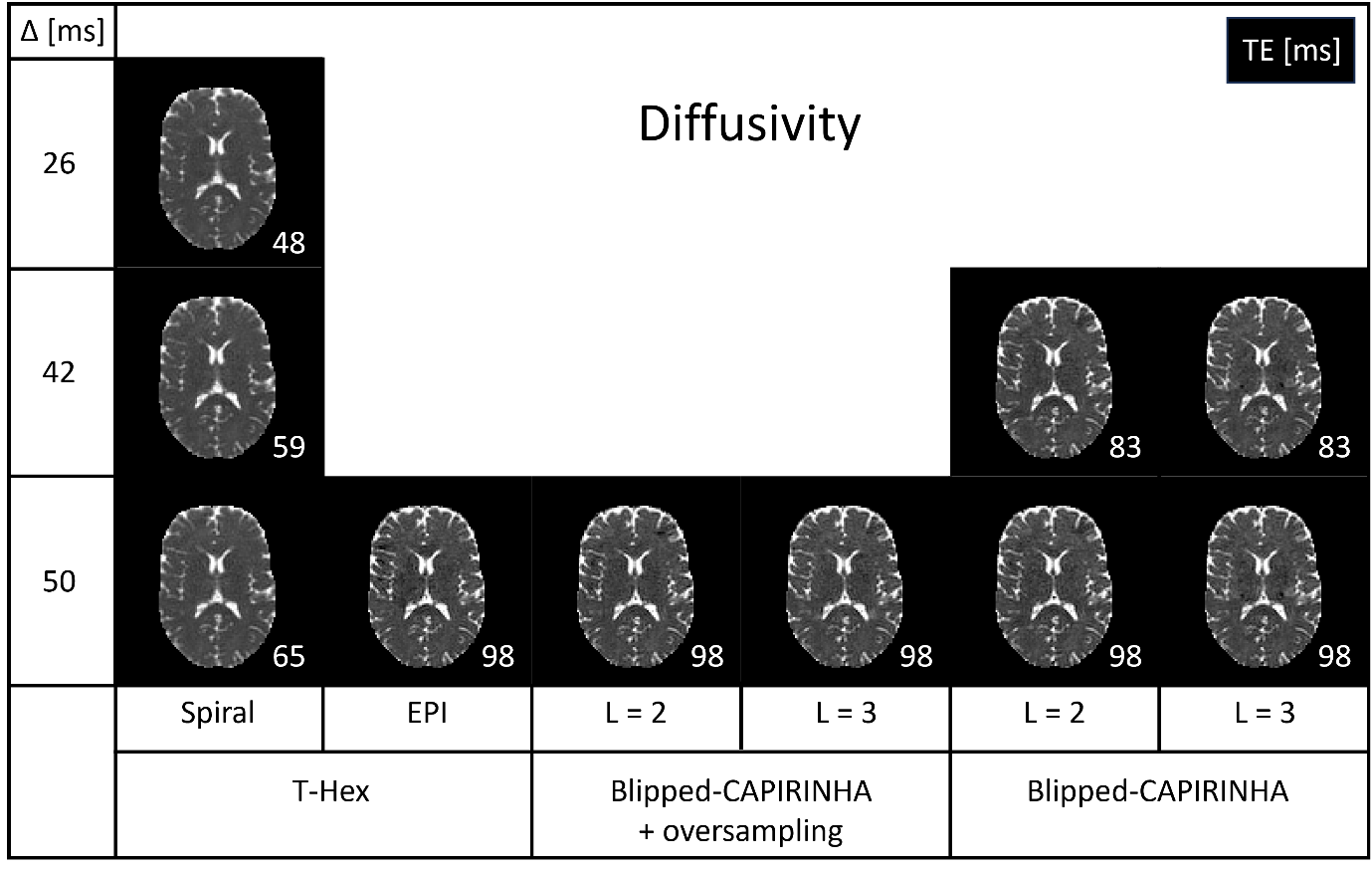


**Figure S4:** Maps of powder average diffusivity for an exemplary transversal slice as obtained from the DKI experiments.


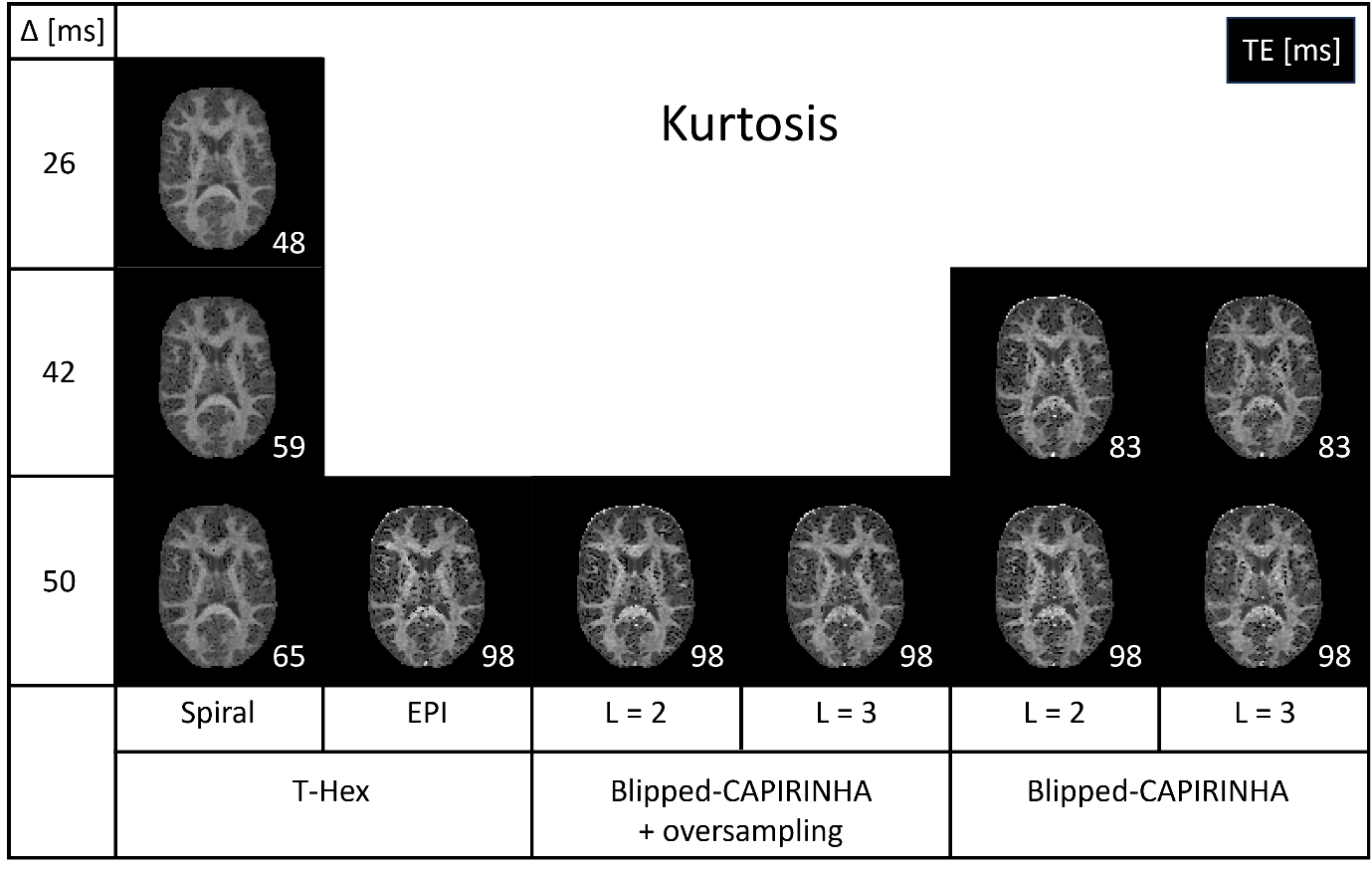


**Figure S5:** Maps of powder average kurtosis for an exemplary transversal slice as obtained from the DKI experiments.


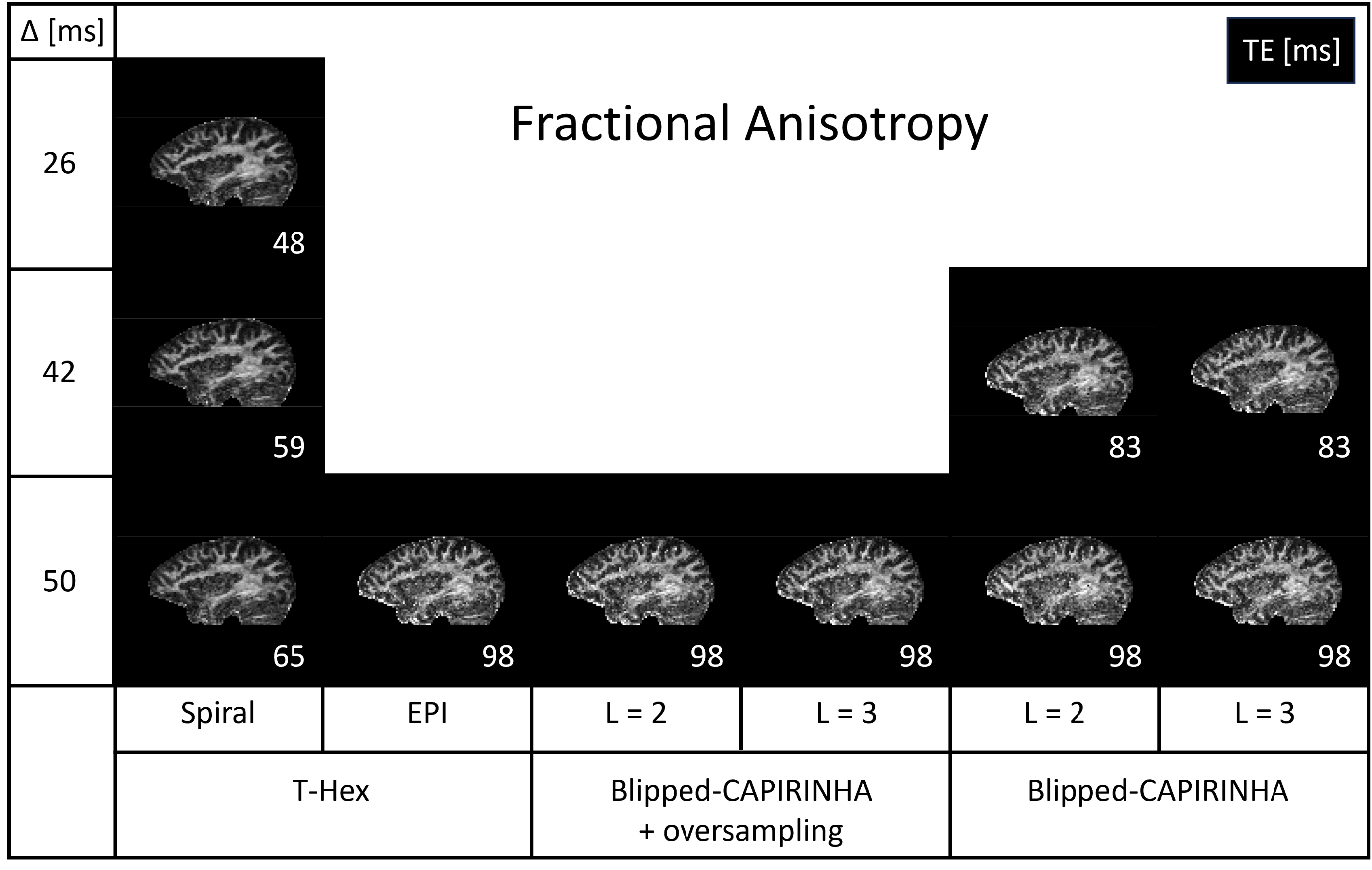


**Figure S6:** Maps of fractional anisotropy for an exemplary sagittal slice as obtained from the DKI experiments.


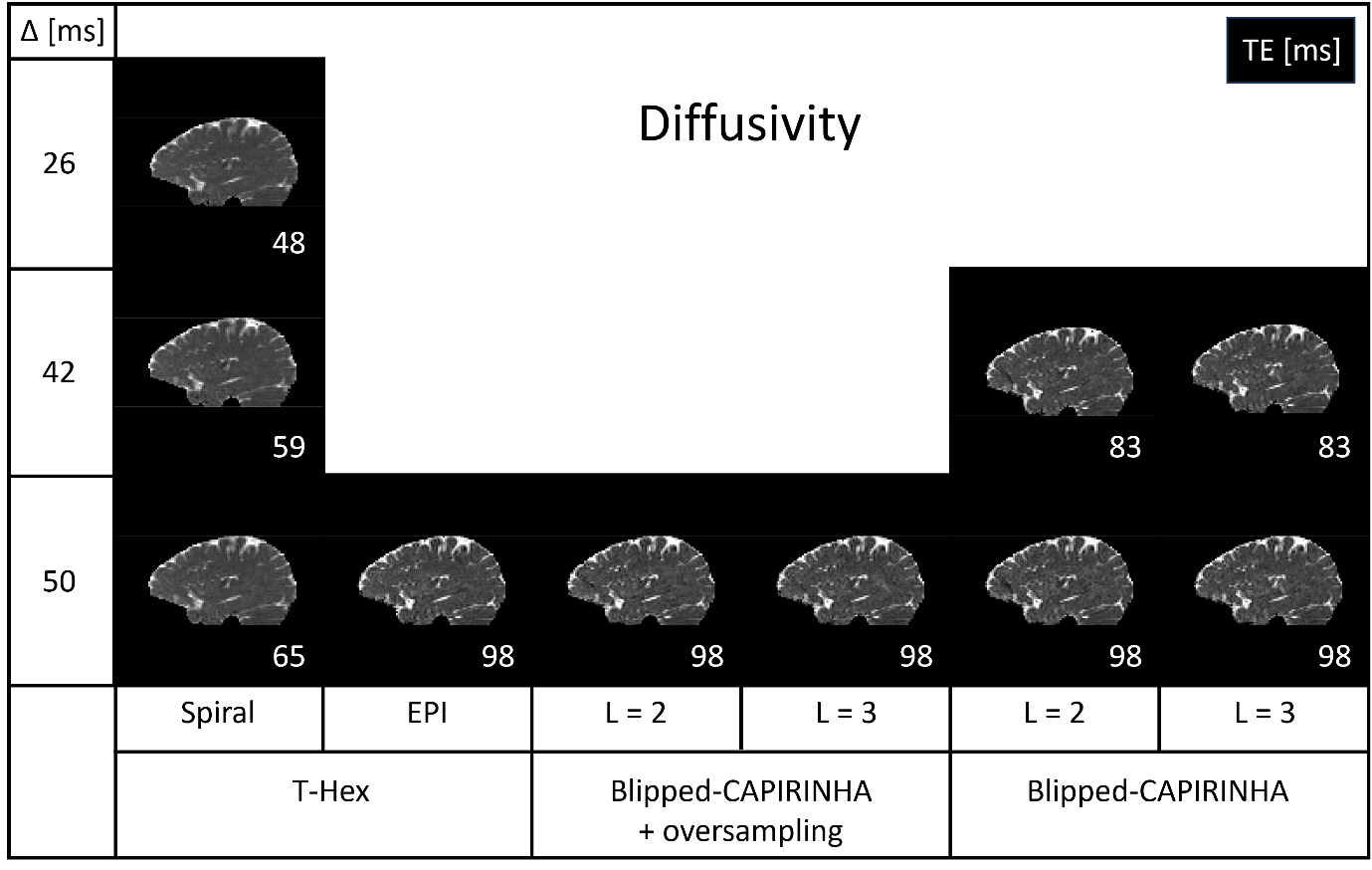


**Figure S7:** Maps of powder average diffusivity for an exemplary sagittal slice as obtained from the DKI experiments.


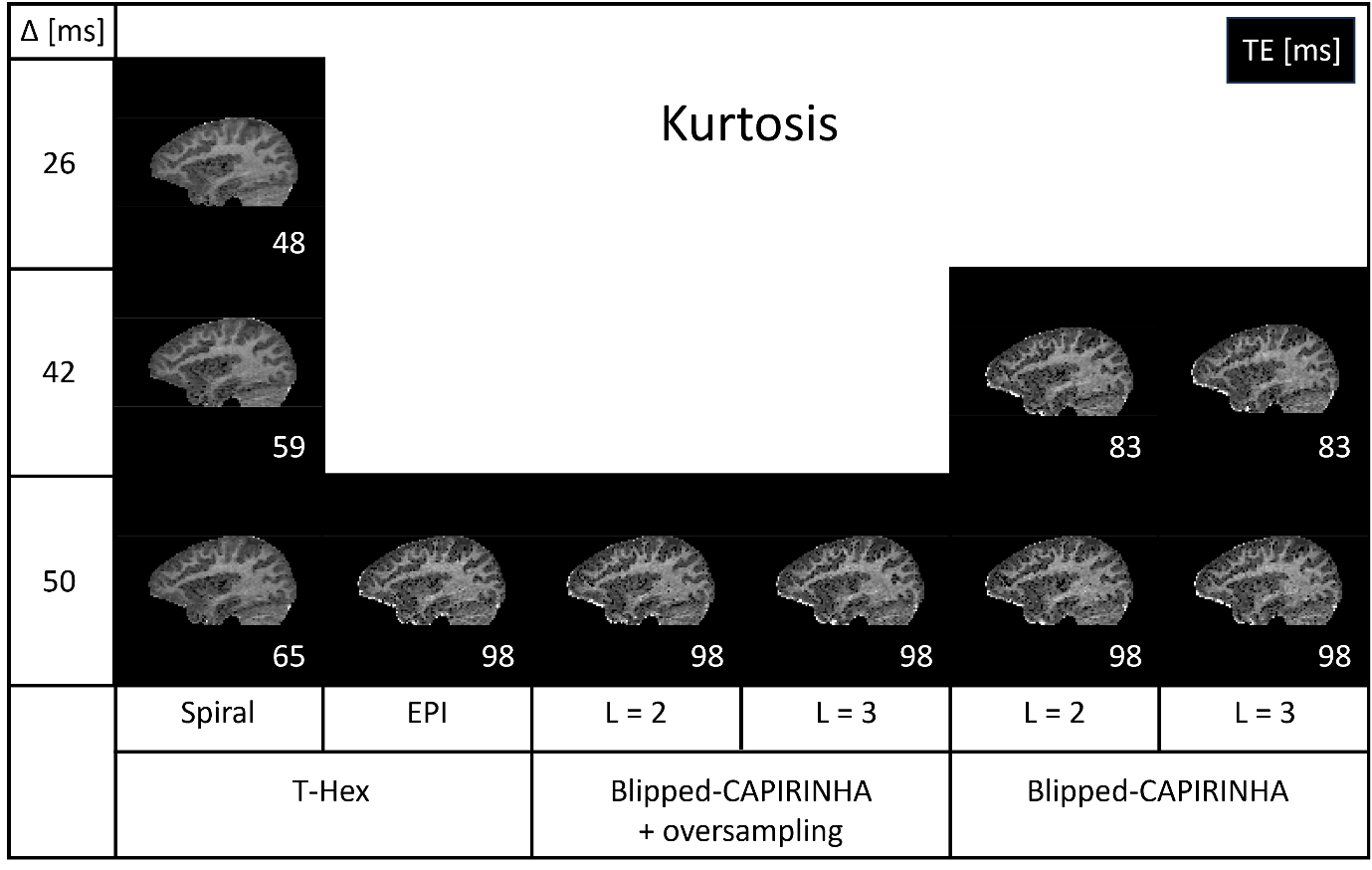


**Figure S8:** Maps of powder average kurtosis for an exemplary sagittal slice as obtained from the DKI experiments.
