# Supplementary figures and images for: Maximizing SNR per unit time in diffusion MRI with multiband T‐Hex spirals
Source: Magn Reson Med. 2023 Dec 29;91(4):1323–36. doi: 10.1002/mrm.29953 (PMC10953427; doi:10.1002/mrm.29953)

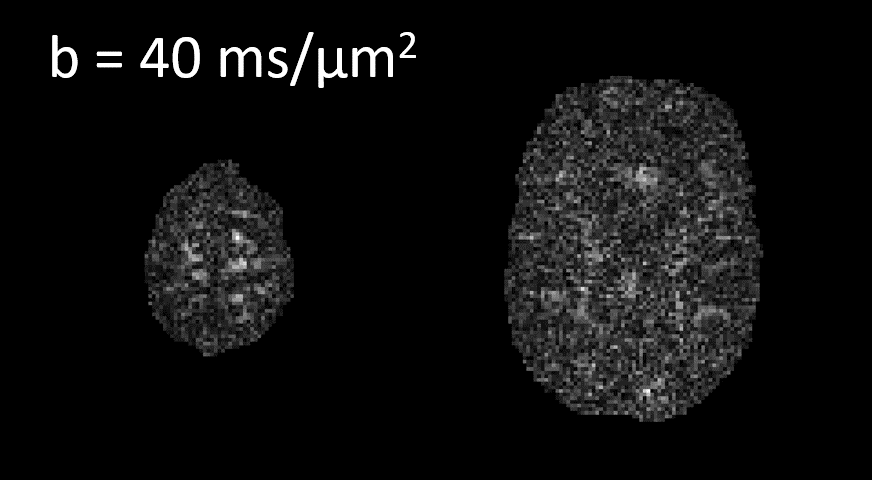

Supplement: Supplementary file 2 — Gif S1. This animation shows the same images as depicted in the rightmost panel in Figure S2 (gray‐scale). The overlay (yellow) is a white matter mask as extracted from an anatomic reference scan. [file MRM-91-1323-s001.gif]
